# De Novo Transcriptome Assembly of Cedar (*Cedrela odorata* L.) and Differential Gene Expression Involved in Herbivore Resistance

**DOI:** 10.3390/cimb46080520

**Published:** 2024-08-14

**Authors:** Luis Felipe Guzmán, Bibiana Tirado, Carlos Iván Cruz-Cárdenas, Edith Rojas-Anaya, Marco Aurelio Aragón-Magadán

**Affiliations:** 1 National Genetic Resources Center, National Agricultural, Forestry and Livestock Researches Institute, Tepatitlán 47600, Jalisco, Mexico; cruz.ivan@inifap.gob.mx (C.I.C.-C.); rojas.edith@inifap.gob.mx (E.R.-A.); 2 Centro Universitario de los Altos, University of Guadalajara, Tepatitlán 47600, Jalisco, Mexico; btiradop@gmail.com

**Keywords:** insect herbivory, infested plant, timber trees, RNAseq, terpene synthase, *Chrysobothris yucatanensis*

## Abstract

Timber trees are targets of herbivorous attacks. The identification of genes associated with pest resistance can be accomplished through differential expression analysis using transcriptomes. We reported the de novo assembly of cedar (*Cedrela odorata* L.) transcriptome and the differential expression of genes involved in herbivore resistance. The assembly and annotation of the transcriptome were obtained using RNAseq from healthy cedar plants and those infested with *Chrysobothris yucatanensis*. A total of 325.6 million reads were obtained, and 127,031 (97.47%) sequences were successfully assembled. A total of 220 herbivory-related genes were detected, of which 170 genes were annotated using GO terms, and 161 genes with 245 functions were identified—165, 75, and 5 were molecular functions, biological processes, and cellular components, respectively. To protect against herbivorous infestation, trees produce toxins and volatile compounds which are modulated by signaling pathways and gene expression related to molecular functions and biological processes. The limited number of genes identified as cellular components suggests that there are minimal alterations in cellular structure in response to borer attack. The chitin recognition protein, jasmonate ZIM-domain (JAZ) motifs, and response regulator receiver domain were found to be overexpressed, whereas the terpene synthase, cytochrome P450, and protein kinase domain gene families were underexpressed. This is the first report of a cedar transcriptome focusing on genes that are overexpressed in healthy plants and underexpressed in infested plants. This method may be a viable option for identifying genes associated with herbivore resistance.

## 1. Introduction

The cedar tree (*Cedrela odorata* L.) is a species of significant importance within its genus due to the quality of its wood and for the biosynthesis of compounds such as triterpenes, monoterpenes, sesquiterpenes, steroids, limonoids, and other meliaceae members, which have potential in medicine and agriculture [1,2].

However, a variety of insects species cause significant damage to tree wood, largely wood-boring beetles are among the main pests affecting forest tree cultivation [3]. In particular, infestation by insects colloquially known as “stem borers” has been observed to reduce the populations of young timber trees [4]. In the case of cedar, it was observed that *Chrysobothris* sp. (from the Buprestidae family) causes mortality rates exceeding 50%, thus qualifying it as an economically significant insect pest [5].

In this context, crop breeding has facilitated the selection and generation of individuals with potential tolerance for biotic stress [6]. Nevertheless, conventional breeding methods require the investment of time, particularly for species with extended life cycles such as trees [7,8]. Consequently, the identification of plant defense mechanisms induced by herbivorous insects represents a strategy to accelerate their genetic improvement using genomic tools [8].

Indeed, insect feeding on plants has been observed to trigger a range of local and systemic signaling mechanisms, which are mediated by transcription factors. These are classified into distinct families, with WRKY being one of the most prevalent and implicated in the growth and response to biotic and abiotic stressors [9]. For example, plants that are tolerant to herbivore attacks exhibit distinct gene expression patterns compared to susceptible plants [10,11,12]. It is noteworthy that the expression of genes in plants in response to stress exhibits variability among individuals and is dependent on a multitude of factors, including genotype, tissue, treatment, time or timing, environmental conditions, and the entire ecosystem [13,14].

For instance, the biosynthesis of jasmonic acid (JA) serves as a primary defense mechanism in plants attacked by herbivorous insect. Similarly, proteins containing the jasmonate ZIM-domain (JAZ) represent a primary target of JA-induced gene expression. Additionally, significant regulation of the AOC (allene oxide synthase), OPR (oxo-phytodienoic acid reductase), ACX1 (acyl-CoA oxidase), and LOX (lipoxygenase) genes has also been observed in poplar affected by beetles [12]. Similarly, Yang et al. [15] observed a significant increase in JA levels and LOX expression, particularly *LOX3*, with positive local and systemic regulations at six and nine hours after herbivory by larvae (*Ectropis oblique*) in tea plants (*Camellia sinensis*), respectively.

A significant observation in tolerant individuals is the overexpression of class I chitinase, which is responsible for the degradation of insect exoskeletons and is associated with direct defense mechanisms in plants. For example, the overexpression of GhChi6 was observed in Arabidopsis (*Arabidopsis thaliana* L.) plants that demonstrated tolerance to aphid infestation [16]. Conversely, in wild pear (*Pyrus betuleafolia* L.) infested with the tobacco cutworm (*Spodoptera litura*), the overexpression of the monoterpene synthase PbeOCS was observed, and its enzymatic product (E)-β-ocimene was found to increase larval mortality [17]. Terpene synthases are indispensable for the synthesis of oleoresin terpenoids, which function as toxic mechanical barriers and are linked to pathogen-induced resistance in conifers. Specifically, in pine (*Pinus massoniana*), the overexpression of *PmTPS4* and *PmTPS21* increased the biosynthesis of oleoresin terpenoids (α-pinene, β-pinene, β-myrcene, D-limonene, and longifolene), thereby enhancing resistance to pine nematode (*Bursaphelenchus xylophilus*) [18].

Another response to herbivore feeding is the activation of the mitogen-activated protein kinase (MAPK) signaling cascade. In this regard, Zhu et al. [19] observed 178 differentially expressed genes in the MAPK signaling pathway, of which *EVM0018807* and *EVM0025267* exhibited a higher expression at the onset and throughout the development of Chinese chestnut (*Castanea mollissima* Blume) shoots infested with gall wasp (*Dryocosmus kuriphilus*). Additionally, alterations in the differential expression of terpenoid biosynthesis, plant hormone signal transduction, and WRKY transcription factors were noted. Despite the variability in plant responses to insect attack, differential expression associated with biological processes may indicate an enhanced defense against insect herbivory [19]. Consequently, the differential expression of genes associated with induced defense mechanisms in pest-tolerant and susceptible plants can facilitate their genetic improvement [20].

Accordingly, the objective of the present study was to assemble the de novo transcriptome of cedar and to identify the differentially expressed genes involved in the defense response against herbivore attack.

## 2. Materials and Methods

### 2.1. Plant Material and RNAseq Sequencing

Plant material was obtained from five healthy cedar plants and six cedar plants exhibiting signs of pest attack by *Chrysobothris yucatanensis.* The plants were sourced from the San Felipe Bacalar nursery of the Chetumal-INIFAP experimental field in Chetumal, Quintana Roo, Mexico. All plants were in their second year of growth, were planted in individual plastic pots, were placed under mesh shade, and were managed equally. Bark samples were collected in 15 mL tubes containing RNAlater (Sigma-Aldrich, Burlington, MA, USA) to prevent transcript degradation until transport to the laboratory. The samples were stored in ultracold storage at −80 °C until RNA isolation was performed.

In the DNA and Genomics Laboratory at the National Center for Genetic Resources-INIFAP, RNA was isolated using the RNeasy Plant Mini Kit protocol (Qiagen, Valencia, CA, USA) in accordance with the manufacturer’s instructions. Quantification was conducted via fluorometry using Qubit 2.0 equipment (ThermoFisher Scientific, Waltham, MA, USA). The integrity of the fragments was verified using nucleic acid electrophoresis using a commercial Bioanalyzer High Sensitivity RNA Analysis method (Agilent, Santa Clara, CA, USA) on a 2100 Bioanalyzer Instrument (Agilent, Santa Clara, CA, USA).

Libraries were prepared with 1 µg of RNA using the TruSeq Stranded Total RNA method (Illumina, San Diego, CA, USA) following the manufacturer’s instructions. The first strand of cDNA was synthesized using SuperScript III Reverse Transcriptase (Invitrogen, Waltham, MA, USA). The quantification and integrity of the libraries were determined via fluorometry using Qubit 2.0 equipment (ThermoFisher Scientific, Waltham, MA, USA) and the commercial Bioanalyzer High Sensitivity RNA Analysis method (Agilent, Santa Clara, CA, USA) on a 2100 Bioanalyzer Instrument (Agilent, Santa Clara, CA, USA), respectively. The library was subjected to next-generation sequencing (NGS) on a MiniSeq Sequencing System (Illumina, San Diego, CA, USA).

The raw sequencing libraries were submitted to the NCBI database under the project number PRJNA1134724, and a copy of the cedar transcriptome was submitted to NCBI with TSA number GKPXP00000000, as well as to figshare, which is available at https://doi.org/10.6084/m9.figshare.26352232.

### 2.2. De Novo Assembly and Quality Analysis

The quality of the sequencing of libraries was evaluated using the Fastqc v0.12.1 software [20]. Subsequently, adapters and sequences with qualities lower than Q25 were removed using the fastp software [21]. The cleaned libraries were then assembled de novo using the default parameters in Trinity RNAseq 2.2.2 software [22]. The software version used was that which was recommended by the authors and is available in the Docker container.

To evaluate the quality of the assembly, it is advisable to compare the transcriptome with reference genomes [23]. In this instance, given that it is a de novo assembly, an alternative approach based on the search for orthologous genes was adopted. This strategy is predicated on the assumption that universal genes are consistently expressed and can be identified as homologs in all organisms [24]. To this end, the BUSCO v 5.4.4 software [24] was used with the transcriptome method (-m transcriptome) and embryophyte lineage (-l embryophyta). An identity value exceeding 80% was indicative of an optimal assembly quality [23,24].

Furthermore, a read support analysis was conducted, and the libraries used in the de novo assembly were mapped using the bowtie2 v2.5.2 software [25], with the “sensitive” flag enabled to enhance the sensitivity in fragment alignment. In accordance with the guidelines set forth by the Trinity RNAseq authors, the mapping values of the reads should exceed 70–80% to guarantee the accurate execution of the assembly.

### 2.3. Redundancy Reduction and Transcriptome Slimming

Transcriptome assemblies and coding sequences are characterized by the presence of numerous artifacts resulting from the assembly process, as well as isoforms, introns, UTRs, and redundant sequences [23,26,27]. Consequently, the additional processes of slimming, which entail the elimination of redundancy and noncoding sequences, are essential.

The elimination of redundancy in transcripts was conducted using CD-HIT v4.8.1 [28] with a similarity identity threshold of 90% (-c 0.9). The resulting data set was then subjected to further processing using TransDecoder v5.5.0 for additional reduction. The parameters for the search of coding sequences (CDSs) and open reading frames (ORFs) were set to proteins with a minimum length of 60 peptides (-m 60). These were then compared using BLASTp [29] and protein families (Pfam) [30].

A bespoke database comprising non-redundant embryophyte protein sequences (UniRef 90) from the UniProt database (consulted in February 2024) was constructed for use in BLASTp. Due to the quantity and complexity of the sequences, the Diamond v2.1.9 software [31] was employed, and an E value of 0.001 (-e 0.001) was used. The Pfam search was conducted using the InterProScan v5.60-92.0 software [32]. The results of the BLASTp and InterProScan analyses were used to retain the aligned transcripts. In TransDecoder v.5.5.0, the options --retain_blastp_hits and --retain_pfam_hits were employed for this purpose. In addition, incomplete peptides from the cedar transcriptome were excluded.

### 2.4. Identification of Herbivore Resistance Genes

Genes associated with herbivore resistance were identified in the slimmed cedar transcriptome through the use of BLASTp. The reference sequences were proteins from the UniProt database that included the keyword “herbivore” and belonged to embryophytes. BLASTp was executed, retaining the top five alignments per sequence and an E value below 0.001. To avoid false positives, alignments with identity values greater than 30% and coverage greater than 50% were retrieved [33].

### 2.5. Differential Gene Expression and Gene Ontology

The sequences obtained through BLASTp were used as a reference transcriptome for differential expression analysis, with a particular focus on the transcriptional variations between these genes in healthy and *C. yucatanensis*-infested plants. This strategy reduces the impact of variations inherent to phenological stages, environmental conditions, and management practices on differential gene expression analysis, thereby facilitating a more accurate identification of genes associated with herbivore resistance [34].

Transcript quantification was conducted using Salmon v.1.9.0 [35]. A differential expression analysis was conducted using the DESeq2 v3.19 software [36] in the R statistical computing environment, version 4.2.2 [37]. The lower limit for the fold change level was set to 1, and p-adj was set to 0.05, as previously described [34,38].

The differentially expressed transcripts were subsequently annotated for GO terms using the InterProScan tool with the—goterms option enabled. WEGO was used to assign terms related to cellular function, biological processes, and cellular components [39].

### 2.6. Validation of Differential Expression

The first strand of cDNA was synthesized from 1 µg of RNA obtained from the vascular cambium of cedar, using the SuperScript III Reverse Transcriptase commercial method (Invitrogen, Carlsbad, CA, USA), according to the manufacturer’s recommendations. The genes terpene synthase (*TERS*), lipoxygenase 1 (*LIPO*1), and mitogen-activated protein kinase (*MAPK*) were selected for the validation of differential expression, and ubiquitin (*UBC*) was included as a reference constitutive gene. Primers were designed using the Primer3 v1.1.4 software, and their specificity was evaluated using the Primer-Blast tool (https://www.ncbi.nlm.nih.gov/tools/primer-blast/index.cgi) accessed on 31 October 2022. The primer sequences are presented in Table 1.

The expression of the TERS, *LIPO*1, and *MAPK* genes was determined using the 2^−ΔΔCt^ relative quantification method using qRT-PCR on a StepOnePlus™ Real-time PCR System thermocycler (Applied Biosystems, Foster City, CA, USA). A total of four samples from healthy plants and two samples from infested plants, respectively, were included in this study. A sample from a healthy plant was used as the control sample. The amplification was conducted in a 20 µL reaction volume, which consisted of 1X Fast Sybr Green Master Mix, 0.2 µM of forward and reverse primers, and 40 ng of cDNA. The amplification conditions were as follows: an activation cycle at 50 °C for 2 min, an initial denaturation cycle at 95 °C for 10 min, followed by 45 cycles of denaturation at 95 °C for 15 s, and annealing and extension at 63 °C, 60 °C, and 60 °C for *TERS*, *LIPO*1, and *MAPK*, respectively, for 60 s. No-template controls were included in each experiment. Gene expression was determined by calculating the Ct values and analyzing the amplification plots obtained using the StepOne v2.3 software.

## 3. Results

### 3.1. De Novo Assembly and Quality Analysis

The de novo assembly of the cedar transcriptome was performed on 11 RNAseq libraries, including 6 from plants exhibiting signs of attack by *Chrysobothris yucatanensis* and 5 from healthy plants. In total, 325.6 million 75 bp reads were used. The transcriptome assembly conducted using Trinity software v2.15.0 yielded 127,031 transcripts and 71,641 genes. The median contig length was 532 pb, the average contig length was calculated at 942.63 pb, and the N50 value was 1623 pb.

A read support analysis using Bowtie2 revealed that 97.47% of the total reads were utilized for the assembly. A functional analysis revealed that 1404 (87.00%) of the 1614 embryophyte-reported genes were complete, as identified by the BUSCO database. Following the elimination of redundancies within the transcriptome, 99,882 unique sequences were recovered, from which 68,617 peptides were obtained. Furthermore, 2698 protein sequences with homology to herbivore resistance genes were identified through BLASTp analysis.

### 3.2. Identification of Herbivore Resistance Genes

**Figure 1:**
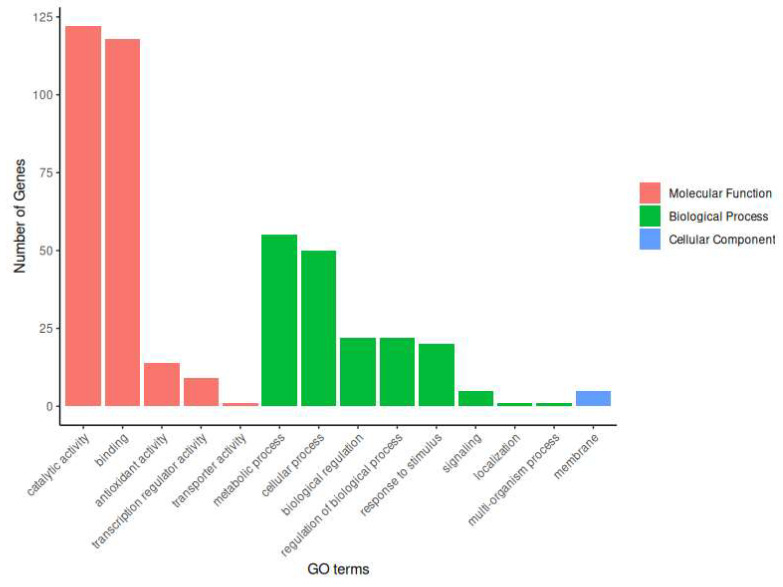
GO term annotation sorted by function.

The putative herbivore resistance genes identified were subjected to differential expression analysis using DESeq2. A total of 220 genes were identified, and all genes were annotated using InterProScan (see Supplementary Table S1). Furthermore, 170 genes were identified with 245 functions, with the most prevalent being molecular functions, biological processes, and cellular components, which were represented by 165, 75, and 5, respectively, through GO terms (Figure 1).

### 3.3. Differential Gene Expression

A total of 161 genes exhibited p-adj values below 0.05, among which 32 demonstrated downregulated expression and 36 exhibited upregulated expression. The annotation of downregulated genes in *C. yucatanensis*-infected plants facilitated the identification of 53 distinct protein families in InterProScan (see Supplementary Material Table S2). Notably, among these families, those related to recognition, signaling, and transcription were particularly prevalent, including the chitin recognition protein, JAZ motifs, and response regulator receiver domain. These proteins are involved in the recognition of chitin in insects and fungi, the regulation of jasmonic acid in response to herbivore attack (JAZ motifs), and the regulation of plant responses in the case of the response regulator receiver domain.

**Figure 2:**
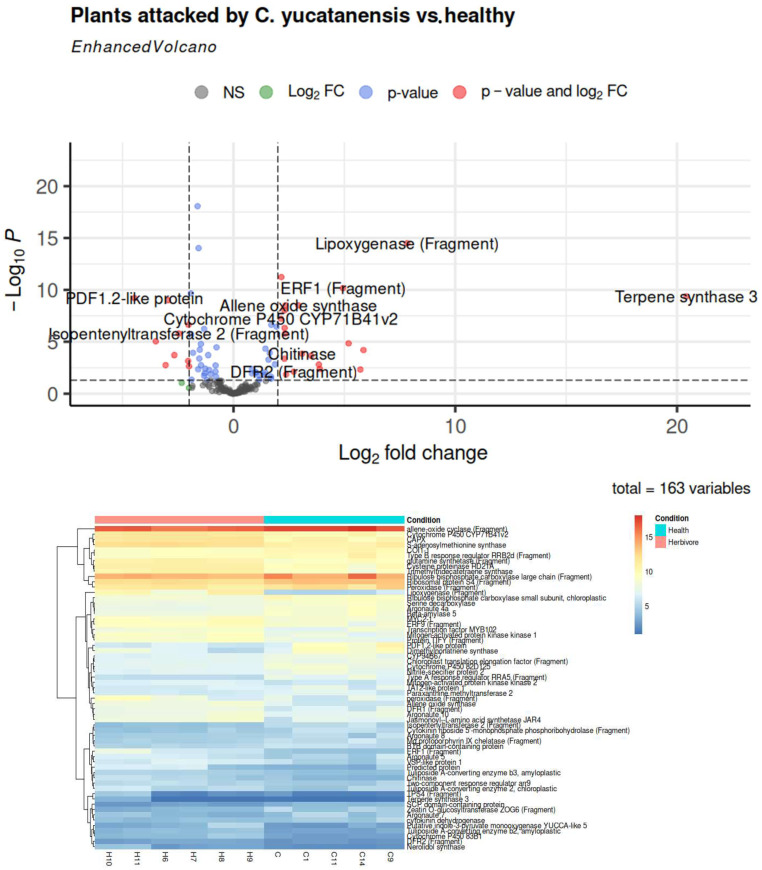
Differentially expressed genes in cedar (*Cedrela odorata* L.) according to DESeq2. (**Up**). Volcano plot; (**down**). Heatmap plot.

In contrast, the annotation of upregulated genes in *C. yucatanensis*-attacked plants identified 41 different protein families, as detailed in Supplementary Material Table S3. These include terpene synthase 3, cytochrome P450, protein kinase domain, chinase, MAPK, NAD-dependent epimerase/dehydratase, zinc-binding dehydrogenase, GroEs-like alcohol dehydrogenase domain, and response regulator receiver domain (Figure 2). The identified families can be classified into three categories—the synthesis of defensive secondary compounds, the signal transduction and regulation of gene expression, and the oxidation and reduction of compounds.

Additionally, domains of defense proteins were identified, including allene oxide cyclase, which is involved in the synthesis of jasmonic acid; lipoxygenase (LIPO1), which is associated with the synthesis of multiple compounds in response to pathogens and wounds; and peroxidases, which are known to be a multigene family with a large number of enzymatic functions, including some related to herbivory responses. Furthermore, terpenoid proteins such as terpene synthase 3 (TES) and cytochrome P450, which are implicated in the regulation of growth and development and functions in the metabolism of organic compounds and herbivore defense, were also identified.

### 3.4. Validation of Differential Expression Using qPCR

**Figure 3:**
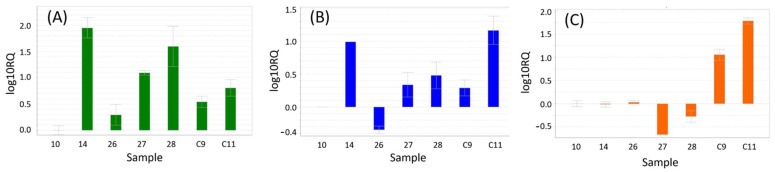
qPCR validation of RNAseq data. (**A**) Expression of the *TERS* gene. (**B**) Expression of the *LIPO*1 gene. (**C**) Expression of the *MAPK* gene. Healthy samples: 14, 26, 27, and 28. Infested samples: C9 and C11. Reference: 10.

The RNA-seq data were validated with the expression patterns of the *TERS*, *LIPO*1, and *MAPK* genes using qPCR. Figure 3 depicts the log10RQ values of four healthy cedar samples and two samples infested with *C. yucatanensis*. The Figure 3 illustrates (A) the overexpression of the *TERS* gene in all samples, (B) the overexpression of the *LIPO*1 gene in five out of six samples, and (C) the overexpression of the *MAPK* gene in infested cedar samples. The un-infested cedar sample was used as a reference, and ubiquitin was employed as the endogenous gene for relative expression. The selected genes exhibited a consistent expression pattern across both according to RNA-seq and qPCR analysis.

## 4. Discussion

The cedar transcriptome was assembled using RNAseq libraries derived from five healthy and six infested plants. A read support analysis of the total reads used for assembly and functionality analysis for gene detection in embryophytes revealed that the assembly meets the required quality standards [22,23,24]. The transcriptome assembly identified herbivore resistance genes that are involved in the defense mechanism against insect attack.

The defense of trees against herbivorous insect attacks is achieved through the production of toxins, defense proteins, or volatile compounds that attract natural enemies [40]. These processes are regulated by signaling pathways and gene expression, which influence the development and survival of herbivores [41,42,43]. These processes are directly related to the molecular functions and biological processes of plants [44,45,46], as evidenced by the higher number of genes annotated in this transcriptome and identified in the present study.

Conversely, the limited number of genes identified as cellular components suggests that alterations in cellular structure are insignificant in response to borer attack. In summary, the response to *C. yucatanensis* attack on cedar was observed at the level of molecular functions and biological processes, whereas the cell structure appears to be unaltered.

The differential gene expression analysis revealed the overexpression of proteins associated with molecular and biological processes. Terpenoid proteins, for instance, play a variety of roles in plants, including the regulation of growth and development, the attraction of pollinators and predators, and defense against herbivores and pathogens [40,41,43,47,48]. Similarly, cytochrome P450, which has a wide range of functions in the plant metabolism of organic compounds, including the synthesis of secondary metabolites, the degradation of toxins, and the biosynthesis of hormones [49]. In the context of plant responses to herbivore attack, the cytochrome P450 complex plays a role in the synthesis of defensive secondary compounds, such as alkaloids, glucosinolates, terpenes, and phenylpropanoids [49,50].

Furthermore, the upregulated gene families identified in this study can be classified according to their primary function. The initial category pertains to the synthesis of defensive secondary compounds, encompassing terpene synthase, cytochrome P450, and potential lysine decarboxylase [47]. The second category is associated with signal transduction and the regulation of gene expression, including NAD-dependent epimerase/dehydratase, protein kinase domain, zinc-binding dehydrogenase, and response regulator receiver domain [51,52,53]. The third category pertains to the oxidation and reduction of compounds, encompassing zinc-binding dehydrogenase and the GroEs-like alcohol dehydrogenase domain [54,55]. These protein families are either directly or indirectly involved in the response of plants to herbivore attack [40,41,44,56].

Transcripts encoding terpene synthase, cytochrome P450, and lysine decarboxylase were observed to be highly expressed (see Supplementary Material Table S4). As previously described, terpenes play various roles in plants, including growth and defense, whereas cytochrome P450 is involved in the metabolism of organic compounds, including the synthesis of defensive secondary metabolites [43]. Additionally, lysine decarboxylase is a pivotal enzyme in the biosynthesis of cadaverine [57], a compound implicated in the plant’s response to biotic and abiotic stress. Furthermore, cadaverine serves as a precursor in the synthesis of quinolizidine alkaloids, which function as secondary metabolites related to plant defense against insects [57,58,59].

In addition, transcripts associated with signal transduction and gene expression regulation, including those encoding NAD-dependent epimerase/dehydratase, protein kinase domain, zinc-binding dehydrogenase, and response regulator receiver domain, as well as transcripts involved in the oxidation and reduction of compounds, including zinc-binding dehydrogenase and GroEs-like alcohol dehydrogenase domain, are present in plants infested by *C. yucatanensis*. However, evidence suggests that herbivore-associated molecular patterns (HAMPs) and effectors in lepidopteran larvae and beetles regulate plant defense responses [60]. HAMPs activate the defense mechanisms of plants, whereas effectors suppress the plant´s responses and increase its susceptibility to subsequent feeding [60].

The differential expression results revealed the reassignment of gene expression associated with molecular defense, arrest, and transcription processes in plants attacked by *C. yucatanensis*. These plants exhibit abundant isoforms of terpene, cytochrome P450, and lysine decarboxylase, which are primarily responsible for the production of secondary metabolites that serve as a defense mechanism against herbivore attack. These findings suggest that the interactions between cedar and herbivores are characterized by dynamic and complex responses involving various molecular and physiological processes. Insect feeding may interfere with the production of stress response hormones, the secondary metabolism of defense proteins, the reassignment of resources, and the modulation of photosystems, which may result in the activation or inhibition of plant defenses. These mechanisms, which have not been extensively studied or understood, have been observed in studies on lepidopterans, in which oviposition reduces the plant’s defense mechanisms to facilitate adequate larval feeding [61,62,63].

The results of this study suggest that the overexpressed genes in attacked plants might be directly related to the defense mechanisms of trees against *C. yucatanensis* and that higher expression could increase the likelihood of resistance to larvae, consequently reducing their attractiveness to lepidopterans as hosts. Thus, focusing on genes that are overexpressed in infested plants and downregulated in healthy plants may represent a better strategy for identifying basal genes related to herbivore defense.

To corroborate the findings of the RNAseq analysis, the expression of three genes in healthy and infested plants was quantified using qPCR. In the course of validating differential expression, the *TERS* and *LIPO*1 genes were overexpressed in both healthy and infested plants, whereas MAPK was overexpressed in infested plants and underexpressed in healthy plants. In order to validate the observed differential expression, the *TERS*, *LIPO*1, and *MAPK* genes were selected based on their functions. Terpene synthases catalyze the production of terpenoid metabolites that are involved in the protection against pathogens, herbivores, and certain abiotic factors [64], as well as in plant growth and development [65]. Lipoxygenases are enzymes that play a role in plant defense against microorganisms, insects, birds, and rodents [66,67]. Mitogen-activated protein kinase (*MAPK*) is involved in regulating a number of important processes in plants, including growth, development, immunity [68], and the plant’s response to various environmental stresses, such as salinity, heat, water deprivation, and biotic stress [69].

## 5. Conclusions

The de novo assembly of the cedar transcriptome was adequate, as evidenced by the high percentage of reads used and the comprehensive annotation of complete genes. This study represents the first report of a cedar (*Cedrela odorata* L.) transcriptome.

The differential expression analysis identified putative herbivore resistance genes that were overexpressed and underexpressed, with molecular functions, biological processes, and cellular components being the most abundant.

A strategy focusing on the genes that are overexpressed in infested plants and those that are underexpressed in healthy plants may provide a more effective strategy for identifying basal genes related to herbivore defense than traditional studies that analyze the genes overexpressed in infested plants.

## Figures and Tables

**Table 1:** Sequences of forward and reverse primers used for amplifying *the TERS*, *LIPO*1, *MAPK*, and *UBC* genes.

Gene	Secuence (5′-3′)		Amplicon Size (bp)
*TERS*	Forward:	TGTGGACTTGAGTTTGCAGC	202
	Reverse:	TCAAAATGCCCTGTGGTGTG	
*LIPO*1	Forward:	GCGTCTCTCATCAATGCAGG	204
	Reverse:	AGCTGGTGGAGGAAAAGTCA	
*MAPK*	Forward:	TAGACAAGGGGCATCCTCTG	150
	Reverse:	CCCGAATGTGATTTCCCTTA	
*UBC*	Forward:	AATCGGAAGAACCGCCATGT	175
	Reverse:	GAACGTACCTCCGTCCCAAG	

## Data Availability

The original contributions presented in this study are included in the article/Supplementary Material; further inquiries can be directed to the corresponding author/s.

## Supplementary Materials

The following supporting information can be downloaded at: https://www.mdpi.com/article/10.3390/cimb46080520/s1, Table S1: Herbivore resistance genes identified in *Cedrela odorata* L. and Pfam and GO annotation; Table S2: Overexpressed genes identified in *Cedrela odorata* L. and Pfam annotation and GO annotation; Table S3: Downregulated genes identified in *Cedrela odorata* L. and Pfam and GO annotation; Table S4: DEG results of infested vs. healthy plants of *Cedrela odorata* L.
